# How Does an Online Mental Health Community on Twitter Empower Diverse Population Levels and Groups? A Qualitative Analysis of #BipolarClub

**DOI:** 10.2196/55965

**Published:** 2024-08-19

**Authors:** Horeya AbouWarda, Mateusz Dolata, Gerhard Schwabe

**Affiliations:** 1 Department of Informatics Faculty of Business, Economics and Informatics University of Zurich Zurich Switzerland

**Keywords:** social media, Twitter, online mental health community, OMHC, empowerment processes, diverse population levels and groups, World Health Organization, WHO, Integrated People-Centred Health Services, IPCHS framework (Strategy 1)

## Abstract

**Background:**

Social media, including online health communities (OHCs), are widely used among both healthy people and those with health conditions. Platforms like Twitter (recently renamed X) have become powerful tools for online mental health communities (OMHCs), enabling users to exchange information, express feelings, and socialize. Recognized as empowering processes, these activities could empower mental health consumers, their families and friends, and society. However, it remains unclear how OMHCs empower diverse population levels and groups.

**Objective:**

This study aimed to develop an understanding of how empowerment processes are conducted within OMHCs on Twitter by identifying members who shape these communities, detecting the types of empowerment processes aligned with the population levels and groups outlined in Strategy 1 of the Integrated People-Centred Health Services (IPCHS) framework by the World Health Organization (WHO), and clarifying members’ involvement tendencies in these processes.

**Methods:**

We conducted our analysis on a Twitter OMHC called #bipolarclub. We captured 2068 original tweets using its hashtag #bipolarclub between December 19, 2022, and January 15, 2023. After screening, 547 eligible tweets by 182 authors were analyzed. Using qualitative content analysis, community members were classified by examining the 182 authors’ Twitter profiles, and empowerment processes were identified by analyzing the 547 tweets and categorized according to the WHO’s Strategy 1. Members’ tendencies of involvement were examined through their contributions to the identified processes.

**Results:**

The analysis of #bipolarclub community members unveiled 5 main classifications among the 182 members, with the majority classified as individual members (n=138, 75.8%), followed by health care–related members (n=39, 21.4%). All members declared that they experience mental health conditions, including mental health and general practitioner members, who used the community as consumers and peers rather than for professional services. The analysis of 547 tweets for empowerment processes revealed 3 categories: individual-level processes (6 processes and 2 subprocesses), informal carer processes (1 process for families and 1 process for friends), and society-level processes (1 process and 2 subprocesses). The analysis also demonstrated distinct involvement tendencies among members, influenced by their identities, with individual members engaging in self-expression and family awareness support and health care–related members supporting societal awareness.

**Conclusions:**

The examination of the #bipolarclub community highlights the capability of Twitter-based OMHCs to empower mental health consumers (including those from underserved and marginalized populations), their families and friends, and society, aligning with the WHO’s empowerment agenda. This underscores the potential benefits of leveraging Twitter for such objectives. This pioneering study is the very first to analyze how a single OMHC can empower diverse populations, offering various health care stakeholders valuable guidance and aiding them in developing consumer-oriented empowerment programs using such OMHCs. We also propose a structured framework that classifies empowerment processes in OMHCs, inspired by the WHO’s Strategy 1 (IPCHS framework).

## Introduction

### The Role of Social Media and Online Mental Health Communities in Consumer Empowerment

In this era of digital interconnectedness, the pervasive use of social media [1], including online health communities (OHCs), has become ubiquitous among both healthy people and those dealing with health conditions [2]. Platforms such as Twitter (recently renamed X) [3,4], Facebook [5], YouTube [6], and Reddit [7] have emerged as powerful tools for the formation and use of online mental health communities (OMHCs), in which users can exchange health-related information, advice, personal experiences, and social support; engage socially; express personal feelings; raise awareness about mental health and influence public conceptions; or merely observe the interactions of others [2,4,5,8-10]. Participating in these “empowerment processes” may lead to significant empowerment outcomes for users of OMHCs [11,12]. This empowerment potential of OMHCs could support various health consumer types, whether they are directly or indirectly engaged with health care systems [13]. It extends beyond mental health consumers (individuals coping with mental health conditions) to include their supporters and informal carers (eg, family and friends) and the broader public. The potential empowerment outcomes for mental health consumers engaging in OMHCs include better health self-management and navigation of health care systems as well as more self-esteem, self-efficacy, and involvement in decision-making during consultations with professionals, which could lead to better health outcomes and quality of life [5,9,14,15]. These online communities could also benefit supporters and informal carers and society at large by enhancing mental health literacy and awareness [16]. However, despite extensive research investigating and acknowledging the pivotal role of social media and OMHCs in fostering consumer empowerment, it has remained unclear how OMHCs empower diverse population levels and groups.

### The Global Consumer Empowerment Movement and the Use of Social Media and OMHCs

Consumer empowerment implies providing people with the educational and supportive resources and opportunities they need in order to acquire the knowledge and skills necessary to have active roles in decisions and actions that impact their health [13,17]. Furthermore, the collaboration between health care stakeholders (eg, organizations and professionals) and health consumers is integral to consumer empowerment in health care systems [13]. This collaboration with consumers, commonly known as “coproduction” and “cocreation” of knowledge generation, decision-making, health services, and healthy environments, plays a crucial role in addressing their specific needs and elevating the overall effectiveness of health care [13,18].

Interest in consumer empowerment has fueled a global health care movement in which individuals, their families, informal carers, and communities are supported to actively participate in decisions and actions that influence their health [13,17,19]. Various health care stakeholders have adopted this movement worldwide, including health systems, authorities, institutions, professionals, and researchers, as well as nongovernmental health organizations, such as the World Health Organization (WHO) [17,19]. Consumer empowerment has become a global choice, as it could not only improve consumers’ health outcomes but could also address the challenges faced by health care systems (eg, rising health care costs, chronic disease burdens, and staff shortages) and contribute to their effectiveness and sustainability [17].

To steer the consumer empowerment movement, the WHO has provided comprehensive guidelines, exemplified by the Integrated People-Centred Health Services (IPCHS) framework, particularly its Strategy 1 (empowering and engaging people and communities), which outlines four key approaches: (1) empowering and engaging individuals and families, (2) empowering and engaging communities, (3) empowering and engaging informal carers, and (4) reaching the underserved and marginalized [13]. In addition, specifically in mental health, the WHO’s Comprehensive Mental Health Action Plan 2013-2030 has embraced consumer empowerment principles [20], emphasizing the importance of empowering and involving mental health consumers, their families, informal carers, and communities in mental health care. These principles are further endorsed in other WHO statements, such as the World Mental Health Report [21] and the Guidance on Community Mental Health Services [22]. In addition to these principles, in the action plan and the other 2 statements, the WHO has encouraged related organizations and professionals to increase the use of social media and digital support groups (eg, online communities) as integral components of digital mental health solutions.

Building on the IPCHS framework’s principles, including its Strategy 1, which suggests the integral role of OMHC users as participants in health systems and aims to provide responsive health services that better address people’s needs both within and beyond the health sector [13], OMHCs could serve as valuable environments for supporting Strategy 1. These online communities could facilitate consumer value cocreation in health care [18,23], in which health consumers from diverse population levels and groups and multiple health care stakeholders (eg, health organizations, professionals, and OMHC moderators) [5,8,10,16,24] could engage in a collaborative process of sharing knowledge, experiences, and insights. These activities could not only benefit consumers within OMHCs but could also enhance the overall quality and responsiveness of online and offline health care services (eg, the development of consumer-oriented empowerment programs in OMHCs and improvements to conventional mental health services). Thus, OMHCs could support the objectives of the IPCHS framework and its Strategy 1, extending beyond traditional mental health service paradigms to promote a more inclusive and accessible consumer empowerment approach. Furthermore, although the WHO’s encouragement to use these platforms may primarily seek to harness such potential advantages, it may also indicate an attempt to fill gaps in mental health services and resources [25] that may not be adequately or readily available elsewhere. This could be due to the challenges within health care systems, such as insufficient funding for alternative mental health solutions. Despite these potential benefits of OMHCs, they are not immune to the drawbacks of online environments. Some studies have reported risks associated with the use of OMHCs, such as the potential of developing unrealistic expectations or confusion about one’s condition due to untrustworthy information from others’ experiences or advice [9] and the possibility of adverse events arising from exposure to triggering content or negative interactions [5]. Nonetheless, despite these risks, the vital role of OMHCs in fostering consumer empowerment cannot be overlooked, given their potential to improve access to mental health resources and address existing service gaps.

Although several health care stakeholders, such as mental health organizations and health professionals [26,27], use OMHCs on social media for consumer empowerment activities, their endeavors cannot be fully realized without a nuanced understanding of how these communities empower diverse population levels and groups. In other words, it is crucial to identify the members who use and form OMHCs, the various types of empowerment processes in such communities, and how their members contribute to these processes. These insights can help various health care stakeholders in leveraging the full potential of OMHCs, enabling the design of tailored supportive resources aligned with specific needs and the development of consumer-oriented mental health programs and services. In addition, a recent study has called for a better understanding of OMHCs on social media regarding how these communities are formed, who their members are, and how they work [5], which underscores the necessity for comprehensive insights to fully leverage their potential and contribute valuable knowledge to the ongoing research in this field.

### Existing Research on Consumer Empowerment Processes in OMHCs and OHCs

Consumer empowerment processes are activities that allow people to be capable, active, and engaged in controlling actions or decisions that impact their health [11,12]. Research in the field of OMHCs and empowerment activities has primarily focused on the empowerment of a specific population level and/or group, including mental health consumers at the individual level [5,15] and those from racial and ethnic minority groups [8]. Some studies were influenced by conceptual frameworks for personal mental health recovery, such as the POETIC (Purpose and Meaning, Optimism and Hope, Empowerment, Tensions, Identity, Connectedness) and the CHIME (Connectedness, Hope and Optimism, Identity, Meaning and Purpose, Empowerment) frameworks [28]. Other studies have concentrated on informal carers and supporters, notably family and friends (pivotal in the well-being of individuals with mental health conditions) [24,29], along with societal-level considerations [16]. These studies indicate that OMHCs on social media involve empowerment processes aligned with the population levels and groups outlined in Strategy 1 of the WHO’s IPCHS framework. However, to the best of our knowledge, existing research has not yet examined these processes in the context of Strategy 1 within OMHCs. In response to the WHO’s call for enhanced use of these online platforms in mental health, gaining deeper insights into empowerment processes aligned with Strategy 1 in OMHCs is crucial to guide effective use. In addition, investigating OMHCs as a digital mental health solution while considering diverse populations and adhering to global standards is of great research interest [30], as it promises to address mental health disparities and enhance global access to care.

Furthermore, current research on empowerment activities has focused mainly on social media–based OHCs addressing various health conditions [31] or web-based OHCs focusing on non–mental health conditions [32,33] rather than specifically investigating social media–based OMHCs. Given the acknowledged differences in the nature of social media–based and web-based OHCs [34] and the variability in support needs across different circumstances [35], extending findings from OHCs not focused on social media and mental health to social media–based OMHCs may not be entirely appropriate. It is also imperative to focus on OHCs dedicated to mental health, given the recent rapid global increase in mental health conditions [36]. This surge in the number of individuals with mental health conditions has put additional strain on health care systems, which may lead, for instance, to longer intervals between consultations. Meanwhile, these individuals may already encounter substantial barriers to using mental health services. For example, recent studies focused on bipolar disorder have reported such barriers faced by those affected, including the delay in obtaining the right diagnosis, limited local availability of psychiatrists, and a lack of insurance coverage [25,37]. Given these challenges, mental health consumers may increasingly turn to OMHCs on popular social media platforms to seek support and connect with peers or professionals, drawing on their familiarity with these platforms from other contexts. Therefore, it is crucial that we urgently examine this specific context.

### Twitter as a Mental Health Solution for Consumer Empowerment

Twitter is one of the most popular social media platforms used in health care practices [34,38]. It offers a distinctive outlet for its users to connect and engage in empowering activities over a popular, publicly accessible, and user-friendly information distribution platform, facilitating the widespread dissemination of information to a wider audience without constraints tied to any account or community following [39,40]. Communities on Twitter are often created bottom-up around a hashtag. Hashtags (a # sign followed by one or more words relating to a specific topic) are usually used to label tweets. These hashtags allow several users to view and contribute to the discussed topic, formulating communities of users talking about common topics, such as #depressionsucks [41] and #schizophrenia [27]. Several studies have reported that mental health–related hashtags on Twitter are valuable solutions for various empowering activities, such as discussing symptoms, personal experiences, life challenges, and mental health care policies; raising awareness; combating stigma; and exchanging different kinds of support [8,16,27,41,42]. Other studies have documented empowerment outcomes for those who use Twitter for mental health discussions [4,43]. Most of these studies have directly and indirectly acknowledged Twitter’s features as conducive to creating a mental health support ecosystem capable of reaching a wide audience, highlighting its potential to support global empowerment initiatives for various populations. However, despite extensive research on Twitter and mental health, the specific processes through which OMHCs on Twitter could empower diverse population levels and groups in line with global empowerment objectives, such as Strategy 1 of the WHO’s IPCHS framework, have not yet been elucidated.

### Purpose of This Study

This study aimed to describe and explain how OMHCs on Twitter empower diverse population levels and groups. Therefore, we formulated three subquestions to address how empowerment processes are conducted within an OMHC on Twitter called #bipolarclub:

1. Who are the members that use and form the OMHC, and how do they participate in it?
2. What are the types of empowerment processes conducted within the OMHC, in accordance with the following population levels and groups outlined in Strategy 1 of the WHO’s IPCHS framework?
  - Mental health consumers
  - Underserved and marginalized mental health consumers
  - Their families and informal carers
  - The broader community (society)
3. What are the tendencies of the OMHC’s members in contributing to and being involved in these empowerment processes?

## Methods

### Overview

To achieve our research goals, we conducted a qualitative exploratory study on the #bipolarclub community. This method offers a nuanced understanding and classification of behaviors, surpassing the scope of statistical analyses. Specifically, we opted for qualitative content analysis because it is a widely recognized and validated method used in previous research to systematically code and categorize communication content on social media platforms [41,44].

Our choice of the #bipolarclub community as the subject of our study is deliberate and aligned with our research context; moreover, this community is active, interactive, substantial, heterogeneous, and rich in its activities, meeting the recommended criteria outlined by Kozinets [45] and Salzmann-Erikson and Eriksson [46] for exploratory studies of online communities. Furthermore, the #bipolarclub community is not merely a congregation around a specific hashtag. It is a well-established entity with a dedicated Twitter account managed by the community’s moderators, who oversee its activities. This characteristic sets it apart as a stable and enduring community rather than one formed around a transient trend.

### Data Collection

We extracted tweets originating from the #bipolarclub hashtag in the Bipolar Club community on Twitter. As evident by its website [47], #bipolarclub is an OMHC that was established in March 2021 by mental health consumers. Despite its name, which suggests a focus on bipolar disorder and a primary aim of supporting those dealing with it, a nuanced perspective is presented on the community’s Twitter account. A prominently pinned tweet clarifies that the community extends beyond a specific disorder or particular persons, welcoming anyone interested in mental health issues generally [48].

**Figure 1:**
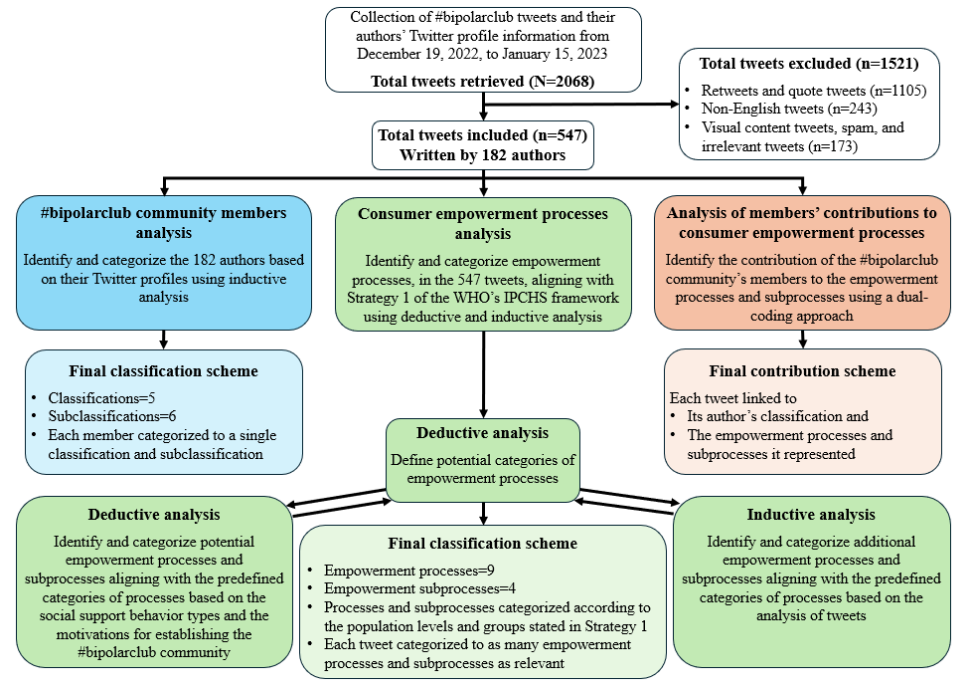
A flowchart of the data analysis process. IPCHS: Integrated People-Centred Health Services; WHO: World Health Organization.

A total of 2068 tweets were collected for a period of 4 weeks from December 19, 2022, to January 15, 2023. We used the Twitter search application programming interface (API; Twitter Inc; now X Corp) [49] and the Postman API development environment (Postman Inc) to gather data end points from Twitter that included the #bipolarclub hashtag. The collected data for each tweet contained its content and the Twitter profile of its author (user’s profile information). From a total of 2068 captured tweets, we excluded some based on the following criteria: (1) the type of tweet was not an original tweet or a reply tweet (retweets and quote tweets, n=1105, 53.4%); (2) the text was not in English (other languages, n=243, 11.8%); and (3) the tweet had no text content or contained irrelevant information (visual content, spam, and irrelevant tweets, n=173, 8.4%). Although tweets should include text content, we also considered embedded media in the analysis, such as images, graphics interchange formats (GIFs), videos, emojis, and links to external websites. After eliminating irrelevant tweets, our final data set consisted of 547 (26.5%) tweets written by 182 authors, as shown at the top of Figure 1.

### Ethical Considerations

Twitter, the platform that hosts the #bipolarclub community, operates as a public platform, allowing its content to be publicly available for research purposes [16]. Notably, only tweets from users with public profiles could be extracted; thus, private profiles are protected by default from being involved in research studies. Given the public nature of the tweets used for the analysis, our study meets the criteria for waiving informed consent [50,51]. Although we met these criteria, we deemed it ethically necessary to inform the #bipolarclub community’s crew about our study intentions beforehand. The first author (HA) communicated with the moderator via private messages from a dedicated Twitter account for the study to the community’s account and clearly identified the researcher’s identity and the study purpose. Subsequently, the moderator discussed our intentions with other crew members and supported our study through a private message on the established communication channel.

Throughout the study, we also adhered to the ethical guidelines of the Association of Internet Researchers [52] to protect Twitter users’ privacy. After the analysis, any information that may have led to the identification of users (eg, @usernames and names) was replaced with pseudonyms in the reported tweets, except for those belonging to institutions that provide mental health care services. The reported tweets were also paraphrased to maintain anonymity, and precautions were taken to ensure that no identifiable information could be retrieved through the Twitter search function or search engines. We ensured that these paraphrased tweets reflect the original tweets’ content. This study’s research protocol and procedures were reviewed and approved by the Human Subjects Committee of the Faculty of Business, Economics and Informatics at the University of Zurich (OEC IRB # 2022-093).

### Data Analysis

#### Overview

After the data collection, all tweets were imported into a secure password-protected Excel spreadsheet (Microsoft Corp) for subsequent qualitative analysis. To facilitate this analysis, we used NVivo software (version 20; QSR International) to conduct qualitative content analysis [53]. The initial analysis was spearheaded by the first author (HA), who developed a preliminary coding scheme to classify #bipolarclub community members and to thematically analyze the empowerment processes conveyed in the tweets. To ensure a systematic approach, hierarchical structures were generated, including classifications and subclassifications for members analysis, along with descriptive categories for empowerment processes analysis. These structures facilitated a standardized comparison among members and across empowerment processes, and they were independently reviewed by the second and third authors (MD and GS) and discussed again, leading to refinement by the entire research team. The 3 researchers who oversaw this analysis have extensive expertise in qualitative research methodologies.

#### OMHC Members Analysis

To identify #bipolarclub community members, we applied an inductive approach to categorize them [53]. We used the user information of the 182 authors of the tweets to inspect their Twitter profiles using the Twitter search function to classify their entities. The categorization was based on cues from their personal descriptions (bios) and the tweets posted on their profiles [54], including pinned tweets as well as tweets posted during the 4-week study period (with or without the hashtag #bipolarclub). However, we only considered their tweets that included the hashtag #bipolarclub to detect their engagement within the community. Each member was assigned to the most pertinent category.

#### Consumer Empowerment Processes Analysis

To detect the empowerment processes aligning with the population levels and groups outlined in Strategy 1 of the WHO’s IPCHS framework in the #bipolarclub community, we analyzed the 547 #bipolarclub tweets using a combined deductive and inductive analysis [53]. Initially, we used 3 frameworks: Strategy 1, social support behavior types, and the motivations of mental health consumers for establishing the #bipolarclub community. This deductive phase laid the groundwork for a categorization structure that encompasses potential empowerment processes.

Empowerment process categories were defined based on Strategy 1 [13], and prior research considered this strategy in mHealth [55], aligning with the population levels and groups stated in the 4 substrategies of Strategy 1: individuals and families (Strategy 1.1), communities (Strategy 1.2), informal carers (Strategy 1.3), and underserved and marginalized individuals (Strategy 1.4). We then used the other 2 frameworks, social support behavior types and motivating factors that led to the community’s establishment, to identify potential empowerment processes and subprocesses aligning with the predefined process categories. The inclusion of social support behavior types stemmed from their prevalence as empowerment processes within OHCs [31]. We also incorporated the motivations that drove mental health consumers to establish the #bipolarclub community because these drivers encapsulate the support needs that these consumers aim to address through this online community, which is a key facet of their empowerment. The social support behavior types were derived from the social support behavior code [32,56,57]. This code encompasses five social support types: (1) informational support, (2) tangible support, (3) esteem support, (4) network support, and (5) emotional support. The motivations for establishing the #bipolarclub community on Twitter were obtained from its website [47]; there were five drives: (1) connecting and making friends, (2) learning from one another, (3) free self-expression, (4) telling personal stories, and (5) fighting stigma. Duplications and overlaps among the 5 social support types and the 5 motivations for establishing the community were addressed, refining the considered empowerment processes for the deductive analysis.

Through an inductive analysis, we then identified additional empowerment processes that derived directly from the data, supplemented by insights from existing empirical and theoretical research. The identified empowerment processes and subprocesses, derived from both deductive and inductive analyses, were systematically organized based on the predefined categories of empowerment processes that align with the population levels and groups outlined in Strategy 1. Each tweet was coded into as many empowerment processes and subprocesses as were relevant, acknowledging that a single tweet may contain multiple statements reflecting various empowerment aspects. This comprehensive coding approach ensured that our analysis appropriately accounted for the multifaceted nature of tweets and the diversity of their content.

#### Examination of OMHC Members’ Contributions to Consumer Empowerment Processes

To identify the tendencies of contribution and involvement of #bipolarclub community members in the empowerment processes and subprocesses within the community, we applied a dual-coding approach. We coded each tweet with (1) its author’s classification code and (2) the empowerment process and subprocess codes it represented. This method enabled us to link the 547 tweets to both their authors’ classifications within the community and the specific empowerment processes and subprocesses to which they contributed. Figure 1 demonstrates the data analysis process in our study to address its 3 research objectives.

## Results

### OMHC Member Analysis

A total of 547 #bipolarclub tweets were written by 182 authors from 13 countries: the United States (n=49, 26.9%), the United Kingdom (n=26, 14.3%), Canada (n=6, 3.3%), Australia (n=3, 1.6%), Norway (n=3, 1.6%), South Africa (n=2, 1.1%), Belgium (n=1, 0.5%), India (n=1, 0.5%), Kenya (n=1, 0.5%), Malaysia (n=1, 0.5%), Russia (n=1, 0.5%), Saudi Arabia (n=1, 0.5%), and Sweden (n=1, 0.5%). However, some users either did not have an available location (n=47, 25.8%) or mentioned a fictional location (n=39, 21.4%).

We categorized the 182 members into 5 main classifications and 6 associated subclassifications. Of the 182 members, the majority were individual members (138/182, 75.8%), followed by health care–related members (39/182, 21.4%), crew and moderator members (3/182, 1.6%), an organizational member (1/182, 0.5%), and the community’s Twitter account (1/182, 0.5%). All of these members, including health care–related members (academics and practitioners), the organizational member (the founder and chairman of the peer support foundation), and those managing the community’s account, were mental health consumers experiencing mental health conditions. This identification was based on statements within their tweets or their Twitter bios, in which they openly shared their experiences of mental health conditions. Furthermore, a key feature of their bios was that many members provided clear insights into their situations as mental health consumers, such as *“*trying to deal with bipolar,” “mental health advocate,” and “Twitter as a venting space.” In addition, several members used usernames and names featuring terms relating to “bipolar” to explicitly express their mental health condition. Classifications and subclassifications of members and their descriptions and proportions of representing the #bipolarclub community are presented in Table 1. Moreover, Multimedia Appendix 1 shows sample paraphrased personal descriptions (bios) and posted tweets on the Twitter profiles of members, offering further clarity regarding their classifications and subclassifications.

**Table 1:** Classifications and subclassifications of #bipolarclub community members (N=182) and their proportions of representing the community.

Classification and subclassifications		Description	Proportion of representing the community, n (%)
**Individual member**		A member who is an individual participant and does not have a health care–related affiliation	138 (75.8)
**Health care–related member**		A member who is an individual participant and has a volunteering, professional, or academic health care–related affiliation	39 (21.4)
	Mental health advocate	A member who is an active mental health advocate that supports promoting mental health care	33 (84.6)
	Mental health advocate and academic	A member who is an active mental health advocate that supports promoting mental health care and has an academic affiliation in the mental health care field (eg, researcher, graduate, and student)	2 (5.1)
	Mental health practitioner	A member who has a professional affiliation in the mental health care field (mental health care service provider)	2 (5.1)
	Mental health advocate and general practitioner	A member who is an active mental health advocate that supports promoting mental health care and has a professional affiliation in the health care field (general health care service provider)	1 (2.6)
	Mental health academic	A member who has an academic affiliation in the mental health care field (eg, researcher, graduate, and student)	1 (2.6)
**Crew and moderator member**		A member who has established and/or run the online community	3 (1.6)
**Organizational member**		A member that is an organizational participant and has a health care–related affiliation	1 (0.5)
	Peer support foundation	A member that provides mental health peer support services as a nonprofit organization	1 (100)
**Community’s account**		A digital member represents the online community’s entity on Twitter, which is run by its moderators	1 (0.5)

The individual members (138/182, 75.8%) who represented the majority of members in the #bipolarclub community were individuals from the general public with no health care–related affiliations. The tweets posted by these individual members primarily revolved around their personal lives and thoughts. The second largest category of members was composed of 21.4% (39/182) of persons with health care–related affiliations, including volunteering (mental health advocates); professional (mental health or general practitioners); or academic (researchers, graduates, or students) affiliations. In this category, members were classified into 5 subcategories. The largest subcategory included mental health advocates (33/39, 85%), who mainly tweeted content that reflected their active advocacy activities for promoting mental health care, such as volunteering for mental health organizations, providing motivational and awareness talks, supporting those who are struggling, and fighting stereotypes and stigma of mental health. It is also worth noting that both practitioner types (mental health and general practitioners) in this community tweeted as mental health consumers and peers, not for professional services, despite being identifiable as professionals in the health care–related classification. Their tweets suggested that they were primarily seeking support from the community. However, they were posting informative tweets to help as peers. This shows how the #bipolarclub community as an OMHC stimulated health care providers to engage as mental health consumers, seeking support rather than using it for professional health care service purposes.

The third category involved crew and moderator members, who represented 1.6% (3/182) of the online community. These members had established the community and/or were running its activities. These activities included retweeting supportive tweets, replying to members, monitoring discussions, posting announcements, contributing with helpful resources, and moderating audio conversations held on Twitter Spaces [58] through the community’s account, which help make the online community a positive outlet for its members [5]. The next classification was formed of an organizational member (1 peer support foundation), representing 0.5% (1/182) of the community. This foundation was a nonprofit organization that provides peer support services. It was tweeting to share its activities, including peer support groups provided by its team members, and to introduce new team members to the community. The last classification consisted of the Twitter account of the #bipolarclub community, with 0.5% (1/182) representing the community’s members. We considered the community’s account as a member in this categorization because it serves as a digital representative of the online community, tweeting and interacting with its members.

### Consumer Empowerment Processes Analysis and Examination of OMHC Members’ Contributions

**Figure 2:**
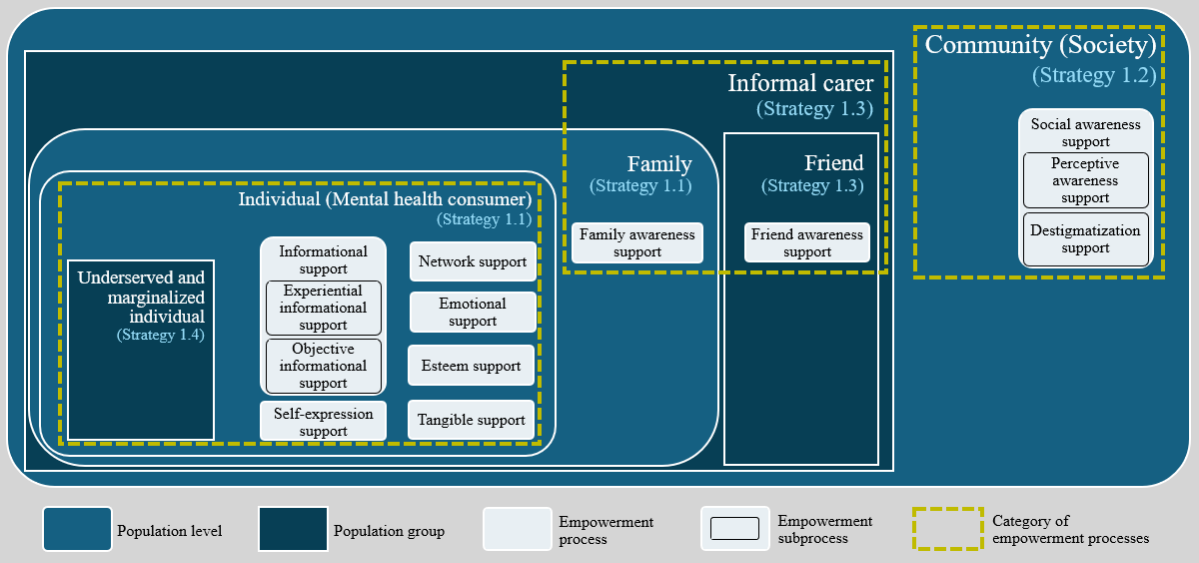
A structured framework of empowerment processes and subprocesses within the #bipolarclub community formulated according to the population levels and groups stated in Strategy 1 of the Integrated People-Centred Health Services framework by the World Health Organization and incorporated the 4 substrategies of Strategy 1 to correspond to each population level and group.

Through the analysis of 547 #bipolarclub tweets, we identified 9 empowerment processes and 4 corresponding subprocesses within the #bipolarclub community. We structured these processes according to the population levels and groups outlined in the WHO’s Strategy 1 (IPCHS framework), along with the substrategy corresponding to each population level and group, as shown in Figure 2.

As illustrated in Figure 2, we positioned mental health consumers at the center, with the underserved and marginalized individuals as a subgroup, encircled by 2 tiers: their supporters and informal carers (primarily their families, followed by their friends) in the inner tier and the broader society in the outer tier. This figure suggests that the #bipolaclub community could foster a supportive and empowering environment, with its influence extending beyond individual well-being to a broader societal impact. As indicated by the 3-dotted boxes in Figure 2, we classified the 9 identified empowerment processes and their 4 associated subprocesses into 3 categories: individual-level processes, informal carer processes, and society-level processes. Individual-level processes support mental health consumers and address the needs of the underserved and marginalized individuals. Informal carer processes extend support to family members and friends, while society-level processes aim to benefit society at large. Our examination revealed that the identified empowerment processes within the #bipolarclub community addressed all the 4 substrategies of Strategy 1. Table 2 provides detailed descriptions, frequencies of tweets, and the contribution proportions of the community members for the identified empowerment processes and subprocesses.

**Table 2:** Empowerment processes and subprocesses detected from #bipolarclub tweets (n=547) aligned with the population levels and groups outlined in Strategy 1 of the Integrated People-Centred Health Services framework by the World Health Organization, a description and the frequency of tweets for each process, and the contribution proportions of the community members.

Population level and group, substrategy of Strategy 1^a^, and empowerment process and subprocess					Description		Sample paraphrased tweet		Tweet frequency, n (%)		Contribution proportions of community members, n (%)
**Individuals (mental health consumers), including the underserved and marginalized individuals**											
	**Strategy 1.1**										
		**Informational support**			Provision of health-related information about mental health conditions and coping strategies, including both objective and fact-based information as well as experiential insights derived from personal experiences		—^b^		519 (94.9)		IM^c^: 331 (63.8) HM^d^: 141 (27.2) CM^e^: 28 (5.4) CA^f^: 17 (3.3) OM^g^: 2 (0.4)
			Experiential informational support	Provision of information derived from personal experiences, offering firsthand insights into navigating everyday life with mental health conditions, as well as self-management and coping mechanisms, including medical, therapeutic, and health care system experiences		“I bought myself a new diary... It helped me track my moods and feelings...”		468 (90.2)		IM: 307 (65.6) HM: 134 (28.6) CM: 27 (5.8)	
			Objective informational support	Provision of information that is impartial and fact based, including mental health–related education materials, advice, and referrals		“...who suffers from bipolar 1 and vestibular migraines, I found this research really interesting...”		113 (21.8)		IM: 53 (46.9) HM: 37 (32.7) CA: 17 (15) CM: 4 (3.5) OM: 2 (1.8)	
		**Self-expression support**			Self-disclosing personal feelings, thoughts, daily life experiences, and challenges of living with mental health conditions, as well as self-motivational expressions		“I’ve never had a life. Only surviving...”		414 (75.7)		IM: 285 (68.8) HM: 106 (25.6) CM: 23 (5.6)
		**Network support**			Communicating with affiliation to the online community as well as provision of offers to gain access to its members		“...We #bipolarclub, could all collectively help to end your depression...”		221 (40.4)		IM: 130 (58.8) HM: 60 (27.1) CA: 17 (7.7) CM: 12 (5.4) OM: 2 (0.9)
		**Emotional support**			Provision of care, love, encouragement, and understanding expressions		“Thank you so much lovely people. Really thanks a lot. I should be dead! But I’m still alive because of you...”		126 (23)		IM: 77 (61.1) HM: 44 (34.9) CM: 5 (4)
		**Esteem support**			Provision of affirmation in ability and compliment expressions, as well as expressions of agreement on a situation and alleviating a sense of guilt about a situation		“It’s great for you, me, and the community that you’re open about how you’re managing your disease. I appreciate the content you’re sharing...”		52 (9.5)		IM: 33 (63.5) HM: 18 (34.6) CM: 1 (1.9)
		**Tangible support**			Provision of offers to help and to join activities or events that are needed to cope with the challenges of mental health conditions, including online and offline peer support groups		“Hello #bipolarclub! Come participate in our Twitter Space gathering today! We’ll be discussing communication skills...”		32 (5.9)		HM: 21 (65.6) IM: 8 (25) CA: 2 (6.2) OM: 1 (3.1)
**Underserved and marginalized individuals (mental health consumers)**											
	**Strategy 1.4**				Provision of diverse forms of individual-level support to address the specific needs of individuals from underserved and marginalized populations, including children and individuals from racial and ethnic minority groups (Black population)		“...@ThinkTenacity...join free Black mental health support group by Black therapists who understand us...”		21 (3.8)		HM: 11 (52.4) IM: 9 (42.9) CM: 1 (4.8)
**Informal carers, including family members and friends**											
	**Strategy 1.1**										
		**Family awareness support**			Provision of information that pertains to both mental health– and family-related aspects, including personal experiences with families		“The past couple of days, I was all right, but today I woke up feeling terrible and can’t bring myself to get out of bed. I hope my daughter can understand...”		39 (7.1)		IM: 27 (69.2) HM: 12 (30.8)
	**Strategy 1.3**										
		**Friend awareness support**			Provision of information that pertains to both mental health– and friend-related aspects, including personal experiences with friends		“...losing friends from having bipolar...it’s hard...no one can understand...”		14 (2.6)		IM: 9 (64.3) HM: 3 (21.4) CM: 1 (7.1) CA: 1 (7.1)
**Community (society)**											
	**Strategy 1.2**										
		**Social awareness support**			Provision of information that pertains to both mental health– and societal-related aspects, addressing social misconceptions and stigmatization of mental health conditions and offering perspectives on the actual experiences and realities associated with mental health conditions		—		77 (14.1)		HM: 36 (46.8) IM: 33 (42.9) CM: 5 (6.5) CA: 3 (3.9)
			Perceptive awareness support	Provision of information that addresses social misconceptions surrounding mental health conditions, including clarifications of these misconceptions, insights into the reality of mental health conditions, and personal experiences in society		“...the most thing I hope people to know about bipolar disorder...that it is not the same thing as mood swings...”		65 (84.4)		HM: 29 (44.6) IM: 28 (43.1) CM: 5 (7.7) CA: 3 (4.6)	
			Destigmatization support	Provision of information that addresses the destigmatization of mental health conditions in society, including antistigma expressions and personal experiences with mental health stigma		“...I discuss bipolar disorder because I want to destigmatize it... Stigma is a terrifying thing...”		18 (23.4)		HM: 12 (66.7) IM: 6 (33.3)	

^a^The 4 substrategies of Strategy 1: Strategy 1.1 (empowering and engaging individuals and families), Strategy 1.2 (empowering and engaging communities), Strategy 1.3 (empowering and engaging informal carers), and Strategy 1.4 (reaching the underserved and marginalized).

^b^Not available.

^c^IM: individual member.

^d^HM: health care–related member.

^e^CM: crew and moderator member.

^f^CA: the community’s Twitter account.

^g^OM: organizational member.

#### Individual-Level Empowerment Processes: Supporting Individuals (Mental Health Consumers), Including the Underserved and Marginalized Individuals

Our analysis of empowerment processes that sought to support individuals with mental health conditions in the #bipolarclub community revealed 6 distinct types of processes and 2 associated subprocesses. This individual-level category of processes could benefit a mental health consumer to be empowered. These processes also addressed the specific needs of the underserved and marginalized individuals. Among the 547 eligible tweets, the 6 identified empowerment processes in the #bipolarclub community included informational support (n=519, 94.9%), self-expression support (n=414, 75.7%), network support (n=221, 40.4%), emotional support (n=126, 23%), esteem support (n=52, 9.5%), and tangible support (n=32, 5.9%). Furthermore, 3.8% (n=21) of the tweets in these processes focused on addressing the specific needs of individuals from underserved and marginalized populations. Table 2 illustrates the results of this analysis, descriptions of processes, samples of tweets, and the contribution proportions of the community members.

The most prevalent individual-level empowerment process in the #bipolarclub community was informational support, constituting 94.9% (519/547) of the tweets. In the informational support process, 2 subprocesses emerged, each defined by the type of mental health–related information exchanged through tweets. Experiential insights from personal experiences shaped the experiential informational support subprocess (468/519, 90.2%), while objective and fact-based information formed the objective informational support subprocess (113/519, 21.8%). Tweets in both subprocesses addressed various aspects of mental health conditions and coping strategies. Experiential informational support (468/519, 90.2%) was the predominant subprocess. In this empowerment subprocess, #bipolarclub community members exchanged personal experiences relating to their daily encounters with mental health conditions, as well as self-management and coping strategies, including medical and therapeutic experiences as well as interactions with the health care system:

...have a space for venting...it helps...

...so happy...first...hospitalization...I feel better...

I have been misdiagnosed over 10 times...

I’ve a Zoom appointment with my psychologist...it’s better to be in person, but he only visits my area monthly...

...if you need support contact @BipolarUK @IntlBipolar...it’s helpful...

...my insurance doesn’t cover my psych meds $1600 a month...

In the other empowerment subprocess, objective informational support (113/519, 21.8%), #bipolarclub community members exchanged tweets involving mental health–related education content, advice, and referrals:

... This article discusses a type of delusion called pseudocyesis, which refers to the false belief of being pregnant...

Take a cozy day off (or three) to recharge...

... Listening to “This Is Bipolar” podcast...

The second individual-level empowerment process was self-expression support. It constituted 75.7% (414/547) of the tweets. Tweets involved in this process show that #bipolarclub community members used it as an outlet for venting and sharing their emotions, thoughts, and challenges of living with mental health conditions, as well as self-motivational expressions:

I am in an absolutely fantastic mood today!...

I feel like I’m losing my fight with my disorder. I feel like my mind is taken a walk off the map...

This week, I’m actively searching for a job. Just last week, I was filling out disability papers. It feels like every week is a guessing game...

I will be okay, it’s not the first time, just like the previous time...

Network support was the third empowerment process in the mental health consumer category, comprising 40.4% (221/547) of the tweets. Members of the #bipolarclub community in this process tweeted expressions of belonging to the online community and exchanged offers to contact one another:

Fellow #bipolarclub...

If you’re up for a chat, please message me. 11:53 PM. I would like a chat with a Peer about right now...

The fourth individual-level empowerment process was emotional support, representing 23% (126/547) of the tweets. This process consisted of tweets among #bipolarclub community members expressing their care, love, encouragement, and understanding to one another:

... To all my #bipolarclub friends and family! Wishing you joyous holidays and hope everyone enjoyed a peaceful day!...

... Thanks #bipolarclub for making this year easier to handle...

... I wish you a wonderful day. If you’re feeling a bit low, just remember you woke up and faced life today. That’s a victory!...

...holidays...I know this time of year can be challenging...

The fifth empowerment process, esteem support, constituted 9.5% (52/547) of the tweets. Tweets in this process revealed that #bipolarclub community members were supporting one another through expressions of positive affirmations regarding capabilities, compliments, agreement on a situation, and alleviation of any feelings of guilt about a situation:

Living with bipolar doesn’t mean you’re broken; it means you are strong and brave for battling your mind every single day...

Yes. I’ve experienced... It’s exhausting...

Completely agree. I’m unable to get help... My general practitioner referrals have been denied twice, and I don’t have the funds for private help...

... If you’re facing depression, know that you’re not alone, millions of people worldwide understand what you’re going through...

The least prominent empowerment process identified in the individual-level category was tangible support, comprising 5.9% (32/547) of the tweets. These tweets demonstrate that the #bipolarclub community was used by its members to offer help to one another and announce activities or events crucial for coping with the challenges of mental health conditions, such as participation in peer support groups:

...I’m here if needed...

Hello #bipolarclub! Join our Twitter Space this Sunday as we delve into a discussion about establishing healthy boundaries in your relationships...

Our analysis also revealed that 3.8% (21/547) of the tweets in the identified empowerment processes at the individual level addressed the specific needs of individuals from underserved and marginalized populations. This formed a distinct subcategory of individual-level empowerment in the #bipolarclub community, as illustrated in Figure 2. The content of tweets in this subcategory was particularly relevant to 2 groups, children and individuals from racial and ethnic minority groups (Black population):

The ACEs (adverse childhood experiences) score is important... Watch this video about childhood trauma!...

...free access to Black therapists...a group support via zoom...meet real Black therapists... Date of event...

As shown in Table 2, #bipolarclub community members from all classifications contributed to the individual-level empowerment processes, with proportions reflecting their representation ratios and classifications within the community. Notably, among the 6 identified empowerment processes, health care–related members were the predominant contributors of tangible support content, in contrast to the other 5 processes with the largest proportion of posts by individual members.

Furthermore, health care–related members made their most substantial contribution to the empowerment process of tangible support, with 66% (21/32) of the tweets. By contrast, both individual members and crew and moderator members demonstrated their highest contribution ratios in self-expression support, with 68.8% (285/414) and 5.6% (23/414) of the tweets, respectively. The community’s account had its most significant contribution rate in the network support process (17/221, 7.7%), whereas the organizational member had it in the tangible support process (1/32, 3%).

#### Informal Carer Empowerment Processes: Supporting Informal Carers, Including Family Members and Friends

The examination of empowerment processes dedicated to supporting informal carers in the #bipolarclub community unveiled 2 processes, one tailored to family members and another to friends. We identified these 2 processes as family awareness support and friend awareness support. Of the 547 tweets, family awareness support comprised 7.1% (n=39) of the tweets, and friend awareness support included 2.6% (n=14) of the tweets. Table 2 shows descriptions of these processes, tweet samples, and the contribution proportions of the community members.

The most prominent empowerment process identified in the informal carer category was family awareness support, constituting 7.1% (39/547) of the tweets. Tweets in this process were composed of information that could support the engagement of families in mental health care, raise their awareness, and allow them to understand their crucial role in the well-being of individuals in their families with mental health conditions. The content of these tweets was related to both mental health conditions and family-related aspects. It involved mental health education materials relevant to families as well as personal experiences shared by #bipolarclub community members. These experiences covered interactions with families in general and specific family members, including parents, children, siblings, and partners:

... Some reading from the Bipolar Disorder Survival Guide “What You and Your Family Need to Know”...

It really hurts being the bipolar child who never gets invited to family gatherings, again...

I find it not easy to discuss my condition with others...especially...my parents...for fear of being blamed; that’s why I can’t be positive, there’re lots of people have rougher lives than you, so on...

My brother led me to a really dark place that I haven’t been in for a very long time, he doesn’t realize how much his words hurt all the time...

I was feeling scattered mentally yesterday, and I was terrible to my partner. I’m so relieved today, and he seems to have forgiven me...

In the informal carer category, the other empowerment process that we identified was friend awareness support (14/547, 2.6%). This process included tweets containing valuable information that could help involve friends in mental health care, enhance their awareness, and help them recognize their significant role in supporting and understanding their friends with mental health conditions. The content in these tweets was relevant to both mental health conditions and friend-related perspectives. It mainly consisted of personal experiences shared by #bipolarclub community members in relation to their friends:

My friend gifted me this wonderful book...it is really helpful during challenging days of depression…

...losing friends from having bipolar...it’s hard...no one can understand...

From the overall contributions of #bipolarclub community members to the 2 empowerment processes of the informal carer category, individual members contributed the most in both processes, family awareness support and friend awareness support. However, the contribution ratio of individual members in family awareness support (27/39, 69%) was higher than in friend awareness support (9/14, 64%). Furthermore, individual members and health care–related members were the only contributors to family awareness support. In addition, health care–related members made their strongest contribution in this category of processes in family awareness support (12/39, 31%). In contrast, the organizational member did not contribute to any process in the informal carer category, while crew and moderator members (1/14, 7%) and the community’s account (1/14, 7%) contributed solely to the friend awareness support process at the same percentage.

#### Society-Level Empowerment Processes: Supporting the Community (Society)

Our analysis of empowerment processes in the #bipolarclub community, aimed at supporting society, indicated that this online community involved 1 overarching process and 2 associated subprocesses. We identified the overarching process as social awareness support and the associated subprocesses as perceptive awareness support and destigmatization support. The findings revealed that of the 547 eligible tweets, social awareness support constituted 14.1% (n=77), wherein perceptive awareness support and destigmatization support accounted for 84% (65/77) and 23% (18/77), respectively. Table 2 describes the processes, samples of tweets, and the contribution proportions of the community members in each process.

The empowerment process of social awareness support (77/547, 14.1%) and its subprocesses, perceptive awareness support (65/77, 84%) and destigmatization support (18/77, 23%), emerged from tweets involving information that could inform and educate the public about mental health conditions and raise their awareness, as well as support to counter stigmatizing attitudes and engage society in mental health care. The provided information pertained to both mental health conditions and aspects associated with society. In addition, notably, tweets in the society-level empowerment processes often featured relevant hashtags such as #MentalHealthAwareness, #BipolarAwareness, #RaiseAwareness, #DepressionIsReal, #MentalHealthMatters, and #BreakTheStigma. This demonstrated that #bipolarclub community members were using their collective voice to reach the public.

The most prevalent subprocess in the social awareness support process was perceptive awareness support (65/77, 84%). This subprocess was composed of tweets containing explanations of misunderstandings regarding mental health conditions, glimpses into the actuality of mental health conditions, and lived experiences of mental health conditions in society:

... Stop saying it’s “their” FAULT to mental patients! The meds disrupt metabolism!...

I’ve been physically fit with a six-pack but still battled major depression and panic attacks...looking good on the outside doesn’t necessarily mean you’re mentally “healthy” on the inside...

I don’t like when people say they’re “so bipolar today” just because they can’t make up their minds...dear, it doesn’t work that way...

I don’t want anyone passing judgment on me during my episodes... Mental health challenges come in various ways. May we all learn to support each other with love, understanding, and compassion...

It seems I have got tardive dyskinesia from my antipsychotics...so embarrassed my twitching is pointed out by my coworker...

The other subprocess, destigmatization support (18/77, 23%), included expressions aimed at destigmatizing mental health conditions as well as personal experiences with mental health stigma:

It isn’t acceptable to use disrespectful, stigmatizing words describing any situation...bipolar is a real mental condition...

...I really want to appear with my real identity to #bipolarclub. But I am scared of the potential of someone from real life finding me. I wouldn’t be able to survive the shame & humiliation again...

While the major contributors in the empowerment processes of the individual-level category and the informal carer category were individual members, health care–related members were the major contributors in the society-level category. Health care–related members contributed to the social awareness support process by 47% (36/77) of the tweets, while individual members participated by 43% (33/77). Furthermore, health care–related members and individual members were the only contributors to the subprocess destigmatization support. In alignment with the informal carer category, the organizational member did not contribute to the society-level category. Crew and moderator members and the community’s account participated in the social awareness support process by 6% (5/77) and 4% (3/77) of the tweets, respectively.

## Discussion

### Principal Findings

#### Overview

In this study, we have investigated an OMHC on Twitter called #bipolarclub by conducting a qualitative content analysis of tweets containing this hashtag circulated between December 19, 2022, and January 15, 2023. Through this analysis, we provided profound insights into three key aspects: (1) the members shaping the online community; (2) the various types of empowerment processes in it, aligned with the population levels and groups outlined in Strategy 1 of the WHO’s IPCHS framework; and (3) the contributions made by its members to these empowerment processes, elucidating their tendencies of involvement.

Overall, we have demonstrated that OMHCs such as #biplolarclub involve health professional members who have dual roles as both professionals and individuals coping with mental health conditions, which highlights the valuable insights and expertise they contribute to the community. We have also revealed that the #bipolarclub community includes empowerment processes catering to all the population levels and groups outlined in Strategy 1 of the WHO’s IPCHS framework. The online community contains processes for mental health consumers, including those from the underserved and marginalized populations, their informal carers (families and friends), and society at large. This finding indicates that an OMHC on Twitter holds promise for empowering diverse populations and supporting global empowerment objectives. Furthermore, our analysis has unveiled distinct contribution tendencies among the members to the empowerment processes in the #bipolarclub community. These tendencies showed diverse patterns of involvement in these processes. In the following sections, we thoroughly discuss our findings and provide suggestions on how these findings can be leveraged to promote consumer empowerment in OMHCs and mental health care.

#### OMHC Members

The #bipolarclub community is formed of 5 primary types of members: individual member, health care–related member, crew and moderator member, organizational member, and the community’s account. This categorization is generally aligned with a previous study that broadly discussed the identities of those who use OMHCs for peer support and the role of moderators in managing the communities [5]. However, our categorization provides deeper insights into the members who form OMHCs on Twitter. All #bipolarclub community members identified with a mental health condition, including practitioner members with health care–related affiliations. However, they used the community as consumers and peers, not for professional service purposes. This finding indicates that the #bipolarclub community involves a unique type of peers that could be identified as “professional peers.” These peers share similar features with those noted in previous studies as formal peers [59] and peer specialists [60]. This peer type in the community provides health-related information based not only on a similar personal experience but also on a professional one. They also serve as linkages with the health system [60].

Furthermore, these peers add value to the #bipolarclub community, as they can bring reliability and credibility to the health-related information provided therein. Compared with a previous study on Reddit, which reported that mental health professionals joined OMHCs solely to offer expert assistance [26], our study shows that professionals may also seek support for themselves as individuals affected by mental health conditions. Thus, we have introduced the concept of “professional peers” as a novel member category in OMHCs on social media that has not been previously documented.

#### Consumer Empowerment Processes

The identified empowerment processes in the #bipolarclub community imply that an OMHC on Twitter has the potential to achieve the empowerment objectives of Strategy 1 of the WHO’s IPCHS framework, along with all its substrategies. This includes empowering mental health consumers as well as addressing the specific needs of the underserved and marginalized individuals, their informal carers (families and friends), and society. The #bipolarclub community comprises 3 categories of empowerment processes, each supporting a specific population level and/or group: the individual-level category (mental health consumers including those from underserved and marginalized populations), the informal carer category (families and friends), and the society-level category.

Regarding the individual-level empowerment processes, the #bipolarclub community involved informational support and its subprocesses, experiential informational support and objective informational support, self-expression support, network support, emotional support, esteem support, and tangible support. Although previous studies indicated that informational and emotional support are the predominant social support types in OHCs [57,61] and are the 2 most common types sought by mental health consumers in online communities [5], our results indicate that the most exchanged social support types in the #bipolarclub community were informational and network support. This finding may be attributed to the networking mechanisms unique to Twitter, such as #hashtags, @username mentions, retweeting, and the following function [40], enabling #bipolarclub community members to engage in more interconnected communication compared with those connected in OHCs on other social media platforms [62]. A prior study that analyzed Twitter hashtags relating to bipolar disorder highlighted emotional support as the predominant social support type [63]. By considering a specific community and the types of members involved therein, we point toward network support as a key aspect. In addition, in a recent study that delved into empowerment processes, “finding recognition” was identified as a process in OMHCs [15]. This process aligns with the network support process in our study, emphasizing the idea that being connected in OMHCs with fellow community members who share similar illnesses and potentially have comparable life experiences can alleviate feelings of loneliness.

Our analysis also unveiled that the experiential informational support subprocess constitutes the majority of its main empowerment process informational support in the #bipolarclub community. This finding suggests that members could primarily use the community to learn from one another their personal experiences of dealing with mental health challenges, which aligns with one of the 5 motivations for establishing the #bipolarclub community (learning from one another) [47]. Although these firsthand experiences can help them in their management and coping strategies [61], they may also lead them to overlook professional guidance. In addition, #bipolarclub community members actively shared information and provided feedback on their experiences with the health care system. This finding indicates that the community could serve a dual function, helping its members to navigate the health care system more effectively [9], as well as offering valuable feedback to health care providers, thereby providing opportunities to enhance health care services [11]. This is particularly relevant in the #bipolarclub community, in which some members have dual roles as both mental health consumers and practitioners.

Our analysis also demonstrated that self-expression support emerged as a frequently practiced empowerment process among #bipolarclub community members. This implies that self-expression may represent a significant need among members, which could be met in the OMHC. Our finding supports previous assertions that mental health consumers commonly use OMHCs on social media [5,9] and Twitter [4] to self-express and vent, reaffirming the notion that OMHCs offer a conducive environment for expressing one’s true self owing to the anonymity afforded by social media [9]. The findings also showed that #bipolarclub community members were expressing their current emotions, thoughts, and challenges instantly, indicating that the real-time nature of Twitter could be a valuable tool for immediate release and a sense of catharsis for those struggling with mental health conditions.

Although the #bipolarclub community generally serves underserved and marginalized individuals (mental health consumers) [64], the content of its tweets addresses the needs of individuals who belong to 2 additional underserved and marginalized populations: children and racial and ethnic minority groups (Black population) [13,55]. The fact that Twitter can support consumers with dual underserved and marginalized status toward empowerment is not novel. An earlier study highlighted the creation of the hashtag #YouGoodMan on Twitter, which is specifically tailored for Black men to share their experiences with mental health conditions, exchange support, and navigate challenges stemming from cultural and social factors in the Black community [8].

Regarding the informal carer empowerment processes, the existence of family awareness support and friend awareness support processes in the #bipolarclub community signifies its potential to empower family members and friends of mental health consumers. The dissemination of mental health–related information linked to families and friends could serve as a valuable resource for enhancing their understanding of the challenges faced by those they are caring for [65,66] and their significant role in their well-being, which could facilitate their active engagement in mental health care practices. In addition, the public nature of Twitter allows users to access a diverse range of perspectives and information. With these findings, we underscore the pivotal role of firsthand information in OMHCs for informal carers, which is sourced directly from individuals who have experienced the situation themselves rather than from other informal carers. This aspect of firsthand information has been overlooked in the existing literature on informal carer empowerment in OMHCs.

Regarding the society-level empowerment processes, the presence of social awareness support and its subprocesses, perceptive awareness support and destigmatization support, highlights the capability of the #bipolarclub community to empower society. The community’s potential role as a facilitator of societal education is apparent through clarifications about social misconceptions regarding mental health conditions, personal experiences in the broader societal context, and antistigma posts. This content could enhance societal understanding and awareness of mental health conditions and could foster societal engagement in mental health care. The destigmatization support subprocess in the #bipolarclub community, aligned with the community’s core objective of fighting stigma [47], could address the stigmatizing challenges encountered by those dealing with mental health conditions in their daily lives in society [67]. Using the #bipolarclub community to combat stigma resonates with previous studies on Twitter [4,68], emphasizing Twitter’s key role in supporting mental health antistigma efforts. In addition, most tweets in the #bipolarclub community centered on raising awareness rather than directly addressing stigmatization. This finding indicates that the community could be adopting a proactive approach to building understanding and empathy with a more inclusive discourse around mental health issues.

#### Contributions of OMHC Members to Consumer Empowerment Processes

When examining members’ contributions to the 3 categories of empowerment processes in the #bipolarclub community, distinct patterns emerged regarding their involvement tendencies, reflecting their identities. Health care–related members were inclined to support societal awareness, mirroring their role as influencers and educators. In contrast, individual members were actively engaged in self-expression and significantly contributed to family awareness, reflecting the importance of personal expression and familial support in this group. In addition, the peer support foundation (organizational member) focused solely on supporting mental health consumers through tangible support, aligning with the core mission of such foundations in providing practical assistance to individuals in need. Akin to individual members, crew and moderator members used the #bipolarclub community as an outlet for personal expression. This tendency conforms with their primary role as individuals coping with mental health conditions, preceding their roles as crew and moderator members. The community’s account stood out for its active role in connecting with members and providing network support, aligning with its identity as a facilitator of community cohesion. These nuanced tendencies underscore the collaborative efforts of different member types, each bringing unique perspectives to the community’s empowerment processes. Notably, our findings differ from those of a study that examined tweets during Mental Health Awareness Week [16], which found similar contribution tendencies to the discourse among diverse users. However, our analysis focused on an OMHC’s tweets, which are not based on a trendy mental health–related hashtag, and we used different criteria for analyzing community members and tweets. Despite differing research goals, these findings suggest that Twitter users’ contribution tendencies in engaging in mental health discussions may vary depending on the context of use.

### Implications

Our study has extended the research on the role of OMHCs in empowering people in significant ways. The key findings are as follows: (1) we identified the diverse members within an OMHC, clarifying their tendencies for engaging in empowerment processes; (2) while health care practitioners use OMHCs to support consumers, we revealed their multifaceted engagement to fulfill various roles simultaneously, acting as mental health consumers, peers, and providers of health information, sometimes disclosing their professional identities; and (3) we proved that an OMHC not only could facilitate empowerment processes for mental health consumers but could also extend their impacts to individuals from underserved and marginalized populations, informal carers (family and friends), and society at large, aligning with the WHO’s empowerment agenda (Strategy 1 of the IPCHS framework). In addition, we have proposed a structured framework for classifying the empowerment processes within OMHCs based on Strategy 1 of the WHO’s IPCHS framework, which embraces individual-level, informal carer, and society-level processes.

The study findings hold significant implications for various health care stakeholders, such as national and international health care organizations, health care professionals, and OMHC moderators. For instance, the WHO and other health care institutions can benefit by learning about the potential of OMHCs for empowering not only mental health consumers but also their families and friends as well as society. Thus, they could consider integrating these communities into standards, acknowledge their important roles in guidelines, and effectively support their development as an element in holistic approaches to mental health care. Our findings can also guide health care organizations and professionals to tailor their interventions and outreach strategies using these communities, ensuring more effective and targeted approaches for diverse population levels and groups to foster empowerment in mental health. Furthermore, moderators of OMHCs can leverage insights from our findings to provide dedicated support for diverse empowerment processes. We speculate that they could explore various uses for initiatives, such as motivating mental health consumers to tell their stories (eg, “tell-your-story” week) to express themselves and raise awareness among others.

### Study Strengths

This study had several strengths. First, our examination of the #bipolarclub community forms part of a larger study to investigate the concept of consumer empowerment in the community following the netnography methodology that focuses on studying online communities’ behaviors [69]. Therefore, the findings of our analysis were based on in-depth immersion in the community. Second, by investigating empowerment processes supporting various population levels and groups, we offer a comprehensive and nuanced view of the empowerment concept within the community. Third, examining empowerment processes aligned with Strategy 1 of the WHO’s IPCHS framework has yielded profound insights into the types of processes that adhere to global standards within OMHCs. Often overlooked in prior studies, this aspect contributes a valuable perspective on the empowerment processes within OMHCs to the existing body of research. Thus, our proposed structured framework of empowerment processes’ classifications within OMHCs, based on Strategy 1, can serve as a novel foundation for future investigations of various OMHCs.

### Study Limitations and Future Research

A possible limitation of this study is the classification of #bipolarclub community members based on their personal bios and the tweets posted on their profiles. This content may not necessarily represent their real-life identity and activities, potentially leading to inaccuracies. However, our passive data collection approach provides real and bias-free insights into how empowerment processes are conducted in the online community [70]. In addition, this approach precluded the examination of a member type in online communities, known as passive members and “lurkers” [71], who may be using the community. Further research could build on our study and explore this member type.

Another limitation of using a passive investigation is the challenge of demonstrating that the #bipolarclub community includes members beyond those who directly experience mental health conditions, such as family members, friends, and the public. However, since our analysis did not include passive members, it is important to acknowledge that merely lurking constitutes a form of engagement in empowerment processes. This implies that these populations still have considerable potential for being involved in the online community. In addition, Twitter’s open-access nature allows not only #bipolarclub community members but also other Twitter users to engage in these processes without restrictions, such as account following [39] or registration requirements such as private groups on Facebook. Furthermore, tweets using the #bipolarclub hashtag were often blended with other hashtags, expanding the community’s reach to a wider audience of Twitter users outside the scope of the community’s members [40], advertently and inadvertently engaging them in the empowerment processes. Future research endeavors could leverage our findings by examining the passive involvement of various population levels and groups within the #bipolarclub community.

We have also primarily focused on one aspect of the consumer empowerment concept, the processes (empowering activities), without considering its other aspect, the outcomes (states of being empowered), which may have limited our findings to provide a comprehensive understanding of the empowerment phenomenon in the online community. However, our study provides in-depth insights into these processes. Future research could investigate the empowerment outcomes in alignment with the empowerment processes we have identified.

### Conclusions

Our analysis of members and empowerment processes in the #bipolarclub community highlights the capability of Twitter-based OMHCs to empower mental health consumers, including those from underserved and marginalized populations, along with their families and friends as well as society. Our study demonstrates the ability of a Twitter-based OMHC to facilitate empowerment processes for diverse population levels and groups aligning with the WHO’s empowerment agenda (Strategy 1 of the IPCHS framework), highlighting the potential advantages of using Twitter for such empowerment objectives. These findings also acknowledge the relevance of Twitter-based OMHCs in advancing global empowerment goals. As the use of OMHCs and Twitter continues to rapidly grow, exploring their potential holds promise for informing various health care stakeholders. This is particularly relevant for health care organizations, professionals, and OMHC moderators, as these insights could pave the way for developing consumer-oriented services and empowerment programs for different population levels and groups.

## Appendix

Multimedia Appendix 1 Sample paraphrased personal descriptions (bios) and posted tweets on Twitter profiles of the #bipolarclub community’s members.

## Abbreviations

API: application programming interface
CHIME: Connectedness, Hope and Optimism, Identity, Meaning and Purpose, Empowerment
GIFs: graphics interchange formats
IPCHS: Integrated People-Centred Health Services
OHC: online health community
OMHC: online mental health community
POETIC: Purpose and Meaning, Optimism and Hope, Empowerment, Tensions, Identity, Connectedness
WHO: World Health Organization
