# Supplementary material for: How Does an Online Mental Health Community on Twitter Empower Diverse Population Levels and Groups? A Qualitative Analysis of #BipolarClub
Source: J Med Internet Res. 2024 Aug 19;26:e55965. doi: 10.2196/55965 (PMC11369525; doi:10.2196/55965)
Supplement: Multimedia Appendix 1 [file jmir_v26i1e55965_app1.pdf]

Multimedia Appendix 1

Sample paraphrased personal descriptions (bios) and posted tweets on Twitter profiles of the #bipolarclub community's members.

Classification and subclassifications		Sample paraphrased bio	Sample paraphrased tweet
**Individual member**		Mother of two, wife, singer, and future cosmetologist. Here for venting!	I was feeling scattered mentally yesterday, and I was terrible to my partner. I'm so relieved today, and he seems to have forgiven me #bipolarclub
**Health care–related member**	Mental health advocate	Mental health advocate. Bipolar and OCD. Blogger and marketing coordinator	Don't miss my next talk on Tuesday, 20 December, at 2:30 PM; if you love this and want more, follow me. #bipolarclub #bipolardisorder #bipolarlife #bipolarawareness #MentalHealthAwareness
	Mental health advocate and academic	Psychologist, postdoctoral @University, and mental health advocate who is living with bipolar disorder	I'm a psychologist. I also write about my experiences of living with bipolar disorder. Mainly, I tweet about research and mental health advocacy
	Mental health practitioner	A psychologist living with bipolar disorder	I have bipolar disorder, but I'm living a normal life. I work as a psychologist. Would you go to a therapist who has bipolar disorder? #mentalhealth #bipolardisorder #therapy

	Mental health advocate and general practitioner	Clinician, wounded healer, and patient	I am a patient first, then a physician who “practices medicine.” This is the art of healing #MedTwitter #bipolar #ptsdawareness
	Mental health academic	BSc Psychology student | Diplomas in cognitive behavioral therapy (CBT) | mindfulness-based cognitive therapy (MBCT) | Bipolar disorder | Eating disorder (ED) recovery | Ballet dancer	No worries, what I did wasn’t a manic impulsive decision #bipolar #bipolarclub
**Crew and moderator member**		I moderate @CommunityAccount. To connect with us and share your stories, utilize the hashtag #bipolarclub. I live with both #bipolardisorder and #borderlinepersonalitydisorder, but neither dictates my identity	Hi #bipolarclub! Check out our Sunday’s Twitter Space group, where we talked about setting boundaries in your relationships! #bipolar #bipolardisorder #mentalhealth
**Organizational member**	Peer support foundation	Peer support institution for bipolar disorder	It is our pleasure to announce some amazing new additions to our Board of Directors! Check us out here on our website #bipolar #bipolarclub
**Community’s account**		All bipolar kids need a place to play. Viewpoints from original authors of tweets. Run by @CrewMember. See the pinned medical disclaimer	Welcome to #bipolarclub! Check out this thread to learn about us!
